# Electronic palliative care coordination systems (EPaCCS): a systematic review

**DOI:** 10.1136/bmjspcare-2018-001689

**Published:** 2019-05-08

**Authors:** Javiera Leniz, Anna Weil, Irene J Higginson, Katherine E Sleeman

**Affiliations:** Cicely Saunders Institute of Palliative Care, Policy and Rehabilitation, King’s College London, London, UK

**Keywords:** electronic health records, advance care planning, palliative care, electronic palliative care coordination systems, EPaCCS, ePCS

## Abstract

**Objectives:**

To systematically search, evaluate and report the state of the science of electronic palliative care coordination systems (EPaCCS).

**Methods:**

We searched CINAHL, MEDLINE, Embase, the Cochrane Library and grey literature for articles evaluating or discussing electronic systems to facilitate sharing of information about advance care plans. Two independent review authors screened full‐text articles for inclusion, assessed quality and extracted data.

**Results:**

In total, 30 articles and reports were included. Of the 26 articles, 14 were ‘expert opinion’ articles (editorials, discussion papers or commentaries), 9 were observational studies (cross-sectional, retrospective cohort studies or service evaluations), 2 were qualitative studies and 1 a mixed-methods study. No study had an experimental design. Quantitative studies described the proportion of people with EPaCCS dying in their preferred place, and associations between EPaCCS use and hospital utilisation. Qualitative, mixed-methods studies and reports described the burden of inputting data and difficulties with IT systems as main challenges of implementing EPaCCS.

**Conclusions:**

Much of the current scientific literature on EPaCCS comprises expert opinion, and there is an absence of experimental studies evaluating the impact of EPaCCS on end-of-life outcomes. Given the current drive for national roll-out of EPaCCS by 2020, it is essential that rigorous evaluation of EPaCCS is prioritised.

## Introduction

Advance care plans allow individuals to specify their wishes and preferences for treatment and care, and have been proposed as a quality indicator for end-of-life care.^1^ For advance care planning to impact maximally on patients and their caregivers, plans need to be made available to all relevant health professionals, including out-of-hours and primary care services, so that care can be delivered in line with patients’ preferences, in a coordinated manner.^2 3^

In the UK, there has been a policy focus on end-of-life care coordination since the Department of Health’s 2008 End of Life Care Strategy, which recommended the creation of Locality Registers for people approaching the end of their life.^4^ Electronic palliative care coordination systems (EPaCCS) emerged to support this. EPaCCS are electronic registers that aim to facilitate documentation of up-to-date information about patients’ preferences and plans for care, in a format that allows sharing of information among different healthcare providers including general practitioner (GP) practices, out-of-hour services, emergency departments, ambulance services, hospices and care homes, among others.^5^ Although many countries have developed electronic palliative care registers, the focus on coordination of care between different settings appears unique to the UK.^5^ EPaCCS started as eight pilots commissioned by the Department of Health in 2009–2011.^6 7^ By 2013, 175 (82.9%) of clinical commissioning groups (CCGs) had either implemented EPaCCS or started planning for their implementation.^8^ In 2015, the Department of Health’s National Commitment for End of Life Care recommended national roll-out of EPaCCS by 2020.^9^

A discussion paper published in 2016 highlighted challenges associated with implementation of EPaCCS and identified key drivers for their success.^5^ This review noted a lack of rigorous research around EPaCCs though this was not systematically synthesised. Our aim was to systematically search, evaluate and report the state of the science of EPaCCS, in order to identify gaps in the evidence and make recommendations for policy and research.

## Methods

### Study design

A systematic review of the state of the science of EPaCCS. The review was conducted and reported following Preferred Reporting Items for Systematic Reviews and Meta-Analyses guidelines.^10^

### Eligibility criteria

#### Study design

All published articles were considered, including editorials, narrative summaries, observational, experimental and qualitative studies. Government or independent reports were included if they reported an evaluation of EPaCCS and contained data not previously published. Conference abstracts were not included.

#### Participants

All studies were included, regardless of participants’ age or disease.

#### Interventions

We included any type of electronic system that enables professionals to share information about advance care plans or advance directives. The rationale for the intervention was the coordination and/or sharing of information about advance care plans between different healthcare providers. Studies describing electronic systems that have no relation to advance care plans or advance directives, or reporting aspects of electronic systems other than sharing or coordination of information (eg, for use by single providers), were excluded. As EPaCCS are a UK initiative, we included only articles describing UK tools.

### Search strategy

The electronic databases searched were: CINAHL (1981 to January 2019), MEDLINE (R) In-Process (1946 to January 2019, via OVID), Embase (1974 to January 2019, via OVID) and the Cochrane Library (up to January 2019). All searches were carried out on the 11 January 2019. Search terms including subject headings and free-text words were developed and adapted for each database (online supplementary file 1). Reference lists of relevant papers were handsearched to identify additional studies. Grey literature including government and independent reports was identified from the references lists of included papers and a simple Google search. Unpublished literature was not identified.

10.1136/bmjspcare-2018-001689.supp1 Supplementary data

### Selection of studies

One of the authors (JL) screened all titles and abstracts retrieved from the electronic database and reference list searches and excluded all those that were not from the UK. Two authors (JL and KES) independently screened the remaining titles and abstracts, and disagreements were resolved by discussion. Full texts were screened by two authors independently (JL, KES or AW). Disagreement was resolved by discussion or consulting a third author. Because we only included UK-based studies, language restrictions were not relevant.

### Data extraction

Data were extracted from all included studies using a data extraction spreadsheet. Data items included study design, aim of the study, setting and participants, the outcome evaluated and results (online supplementary file 2, table 1). Two authors (JL, AW or KES) independently extracted data on all studies and disagreements were resolved by discussion or in consultation with a third author (KES or AW).

10.1136/bmjspcare-2018-001689.supp2 Supplementary data

**Table 1:** Characteristics of included studies

Author	Study design	Level of evidence	Qual/quant	Region	Participants	Methods of data collection	Type of outcome reported
Ali *et al* 28	Retrospective cohort	3. Controlled cohort and case control studies	Quantitative	Grampian	Patients with diagnosis of cancer and ‘terminal care’ who contacted the GMED from January 2011 to December 2011	Secondary data analysis	Admission to hospital and length of stay
Allsop^15^	Commentary	6. Expert opinion	NA	NA	NA		NA
Allsop *et al* 6	Retrospective cohort	4. Qualitative and observational without control group	Quantitative	Leeds	Patients who died from April 2014 to March 2015 with an EPaCCS record	Secondary data analysis	Achievement of PPD and ACP documentation
Broadhurst *et al* 29	Retrospective cohort	4. Qualitative and observational without control group	Quantitative	London	Patients with an EPaCC created between December 2015 and September 2016	Secondary data analysis	Achievement of PPD
Callender *et al* 30	Retrospective cohort	4. Qualitative and observational without control group	Quantitative	London	Patients who died from March 2011 to September 2016 with an EPaCCS record	Secondary data analysis	Achievement of PPD
Hall *et al* 35	Qualitative interview study	4. Qualitative and observational without control group	Qualitative	Grampian and Lothian	Primary care and out-of-hours healthcare professionals, patients and carers users of EPaCCS	22 semistructured interviews	Implementation
Hamilton^16^	Editorial	6. Expert opinion	NA	NA	NA		NA
Henry and Hayes^17^	Commentary	6.Expert opinion	NA	NA	NA		NA
Hunt^18^	Commentary	6.Expert opinion	NA	NA	NA		NA
Johnson^19^	Commentary	6.Expert opinion	NA	NA	NA		NA
Jones and Whitmore^20^	Editorial	6. Expert opinion	NA	NA	NA		NA
Lindsey and Hayes^21^	Commentary	6. Expert opinion	NA	NA	NA		NA
Millares Martin^31^	Cross-sectional	4. Qualitative and observational without control group	Quantitative	UK	CCGs across England	209 surveys	Implementation
Millington-Sanders *et al* 32	Descriptive	4. Qualitative and observational without control group	Quantitative	Richmond, London	Patients with an EPaCCS record between November 2010 and August 2012	Secondary data analysis	Achievement of PPD
Mullick *et al* 22	Narrative review	6. Expert opinion	NA	NA	NA		NA
Murphy-Jones^23^	Commentary	6. Expert opinion	NA	NA	NA		NA
Petrova *et al* 5	Discussion paper	6. Expert opinion	NA	NA	NA		NA
Pringle *et al* 33	Service evaluation	4. Qualitative and observational without control group	Quantitative	Lothian	Patients known to specialist palliative care community team	Secondary data analysis	ACP documentation
Purdy *et al* 34	Retrospective cohort with control group	3. Controlled cohort and case control studies	Quantitative	North Somerset and Somerset	Patients who died from September 2011 to February 2012 and potentially eligible for EoLC	Secondary data analysis	Place of death and hospital admissions
Riley and Madill^24^	Discussion paper	6. Expert opinion	NA	NA	NA		NA
Sleeman and Higginson^25^	Commentary	6. Expert opinion	NA	NA	NA		NA
Smith *et al* 7	Retrospective cohort	4. Qualitative and observational without control group	Quantitative	London	Patients who died from August 2010 to March 2012 with an EPaCCS record	Secondary data analysis	Achievement of PPD
Smith and Riley^26^	Commentary	6. Expert opinion	NA	NA	NA		NA
Wye *et al* 36	Realistic evaluation	4. Qualitative and observational without control group	Qualitative	North Somerset and Somerset	Healthcare professionals, patients and carers users of EPaCCS	Documentation analysis, 15 observations of services, 148 interviews	Implementation
Wye *et al* 37	Mixed-methods	4. Qualitative and observational without control group	Mixed methods	North Somerset and Somerset	Healthcare professionals users of EPaCCS	101 interviews	Implementation
Wye^27^	Commentary	6. Expert opinion	NA	NA	NA		NA

ACP, advance care planning; CCG, clinical commissioning group;EPaCCS, electronic palliative care coordination systems; EoLC, end-of-life care; GMED, Grampian medical emergency department; NA, not applicable; PPD, preferred place of death.

### Quality evaluation

The quality of the included articles was assessed using the Standard Quality Assessment Criteria for evaluation of primary research papers from different fields and design.^11^ The checklist for assessing the quality of quantitative articles consists of 14 potential criteria scored on a 3-point scale (higher points indicate better quality). The quality assessment checklist for qualitative studies is scored in a similar way and consists of 10 essential criteria. Two authors (JL, AW or KES) independently appraised the included articles. Disagreements were resolved by discussion or in consultation with the third author. Reports were not included in the quality assessment. All eligible studies were included in the review, irrespective of quality score.

### Analysis

We derived a hierarchy of evidence incorporating both quantitative and qualitative studies, adapted from primary work by Murad *et al*.^12^ We included editorials, commentaries, discussion papers and narrative summaries as ‘expert opinion’. Reports were not included in the evidence hierarchy but reported separately. References were managed using EndNote V.X7,^13^ and data were tabulated using Excel.^14^ A descriptive summary of results was reported.

## Results

**Figure 1:**
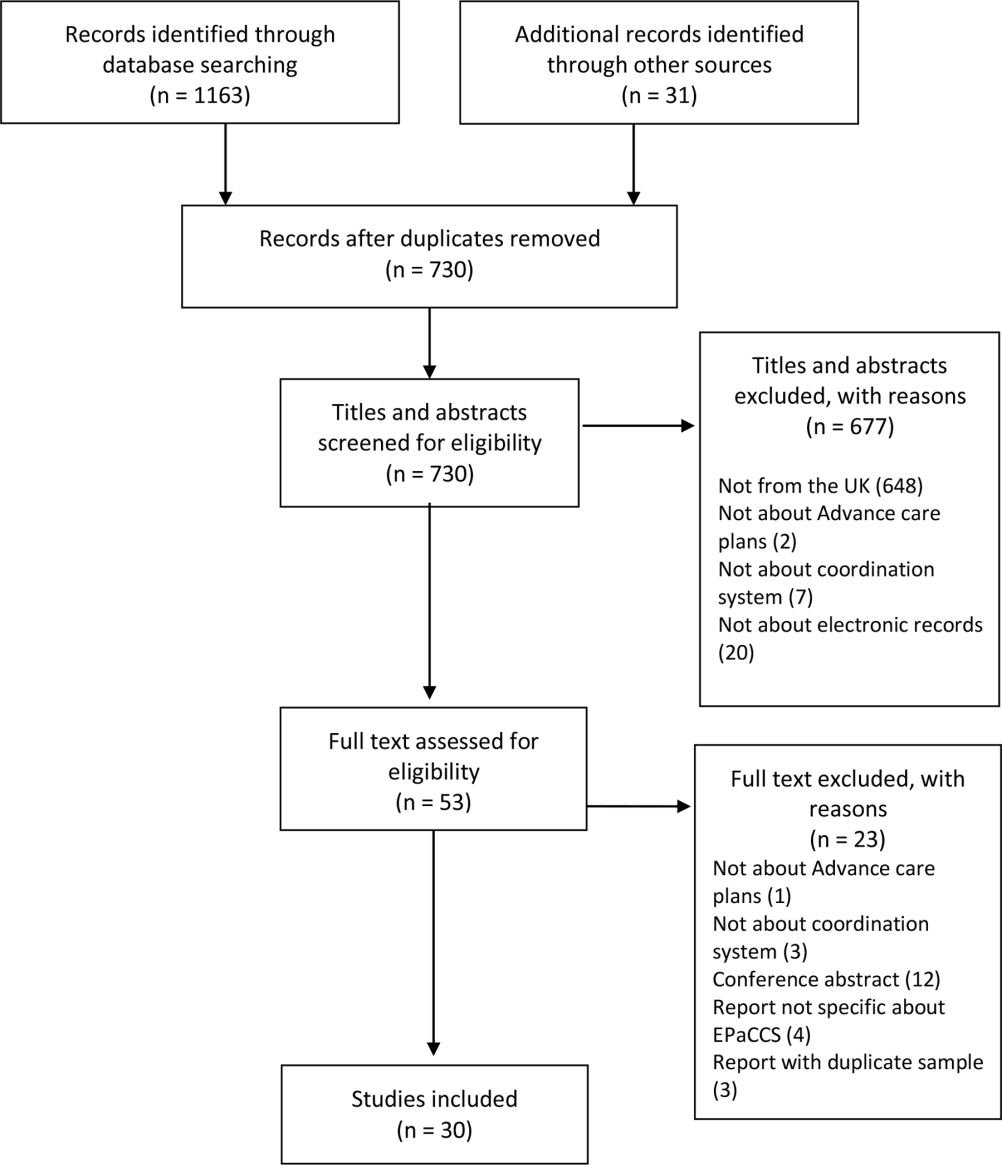
PRISMA flow diagram if papers reporting numbers of included and excluded studies. EPaCCS, electronic palliative care coordination systems; PRISMA, Preferred Reporting Items for Systematic Reviews and Meta-Analyses.

The search strategy identified 1163 articles. Thirty-one additional articles and reports were identified from handsearching. After removing duplicates, 730 titles and abstracts were screened. Six hundred and seventy-seven articles were excluded based on the title or abstract. Fifty-three relevant full texts were screened. Twenty-three were excluded because they were not about advance care planning, not about coordination systems, were conference abstracts or were reports with data reported in another article already included. Table 2 in the online supplementary file 2 reports characteristics of excluded studies. Twenty-six studies and four reports were included in the qualitative synthesis (figure 1).

**Table 2:** Characteristics of included reports

Author	Study design	Level of evidence	Qual/quant	Region	Participants	Methods of data collection	Type of outcome reported
Ipsos MORI^39^	Pilot evaluation	Report	Qualitative	Brighton and Hove, London, Leeds, Mid Essex, Salford, Sandwell and North Somerset	Pilot leaders	Document analysis, interviews	Implementation
NHS Improving Quality^38^	Cross-sectional	Report	Quantitative	CCGs in England	CCGs across England	188 surveys	Implementation, ACP documentation and place of death
NHS Improving Quality^40^	Mixed methods	Report	Quantitative	Brighton and Hove, London, Leeds, Mid Essex, Salford, Sandwell, Medway, Bedfodshire, Birmingham, North East and North Somerset	HES hospital care, local EPaCCS data and ONS data on DiUPR from January 2008 to June 2012. Members of EPaCCS team.	Secondary data analysis and 55 surveys	DiUPR, achievement of PPD, hospital admissions, cost and implementation*
			Qualitative		EPaCCS team members	Two focus group	Implementation
Whole System Partnership^41^	Mixed methods	Report	Quantitative	10 sites from London, South West England, East of England, North West and East Midlands	ONS data on place of death in 139 CCGs between 2011 and 2016. HES data on hospital admissions in the last year of life in 10 CCGs areas.	Secondary data analysis and 91 surveys	DiUPR and cost
			Qualitative		Healthcare professionals, patients, carers, system leaders.	12 in-depth interviews	Implementation

*Data on place of death were only available for four EPaCCS sites.

ACP, advance care planning; CCG, clinical commissioning group; DiUPR, death in usual place of residence;EPaCCS, Electronic Palliative Care Coordination Systems; HES, Hospital Episodes Statistics;NHS, National Health Service; ONS, Office of National Statistics; PPD, preferred place of death.

### Summary of included studies

#### Study design

**Figure 2:**
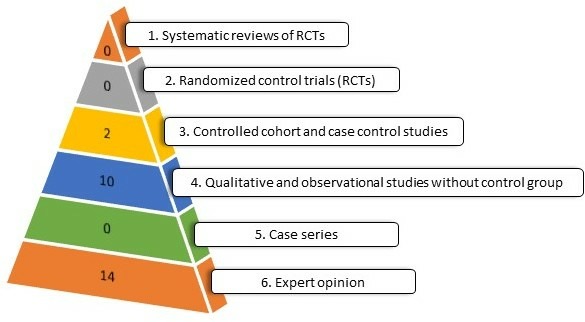
Type of studies included according to the hierarchy of evidence based on Murad et al.^12^

Fourteen of 26 articles comprised ‘expert opinion’ (editorials, commentaries and discussion papers).^5 15–27^ Of the remaining 12 articles, nine were quantitative studies,^6 7 28–34^ two were qualitative^35 36^ and one was a mixed-method study though predominantly qualitative.^37^ All nine quantitative studies had an observational design: one was a service evaluation,^33^ two were descriptive cross-sectional studies,^31 32^ four were retrospective cohort studies without control groups^6 7 29 30^ and two were retrospective cohort studies with control groups^28 34^ (figure 2). All studies using qualitative methods used semistructured interviews,^35–37^ and one used analysis of documentation and service observations in addition to interviews.^36^ Table 1 reports characteristics of included articles.

Of four reports, one was a cross-sectional survey,^38^ one used a qualitative approach through interviews and documentation analysis,^39^ and two used mixed-methods approaches combining both quantitative and qualitative data analysis.^40 41^ Table 2 reports characteristics of included reports.

#### Population

Of the 12 articles including primary data, all included individuals with a terminal condition. One article focused exclusively on patients with cancer,^28^ while the rest did not specify any particular condition. In four articles the population was individuals who had died.^6 7 30 34^ In five articles, the population was patients who were registered on an EPaCCS system.^6 7 29 30 32^ In four of the articles, participants were healthcare professionals^35–37^ or commissioners at the CCGs level.^31^ The four reports included variously EPaCCS leaders,^39–41^ healthcare professionals and patients,^41^ and commissioners.^38^

For four of the articles the setting was London.^7 29 30 32^ Three were from Grampian and Lothian,^28 33 35^ three from Somerset,^34 36 37^ one from Leeds^6^ and one was nation wide.^31^ All four reports included multiple regions in England.

#### Quality appraisal of included articles

The mean quality appraisal score for quantitative studies was 84.8% (online supplementary file 2, tables 3 and 4). The most common source of poor quality for quantitative studies was the lack of an evident and appropriate study design and a poorly defined comparison group. The mean quality appraisal score for qualitative studies was 83.3%. The most common source of poor quality for qualitative studies was a lack of reflexivity of the account and description of the theoretical framework.

**Table 3:** Characteristics of EPaCCS studies reporting place of death and preferred place of death

Author	Sample	% with cancer	PPD home (%)	PPD achieved (%)	PPD achieved if PPD was home (%)/hospice (%)	Likelihood of hospital death OR (95% CI)*
Allsop *et al* 6	1229	–	55	75	65/83	–
Broadhurst *et al* 29	6854	43	55	79	–	–
Callender *et al* 30	9027	57	65	78	72/79	–
Millington-Sanders *et al* ^32^	597	–	35†	–	–	–
Smith *et al* 7	207	46	40	55	68/34	–
Purdy *et al* 34	1022‡	68	–	–	–	0.30 (0.13 to 0.69)*
	2572§		–	–	–	0.22 (0.12 to 0.40)*

*OR for dying at home for those with an EPaCCS record versus those without an EPaCCS record, adjusted by gender, age, deprivation and cause of death.

†29.0% of the 138 individuals who died in the sample died at home.

‡North Somerset.

§Somerset.

EPaCCS, electronic palliative care coordination systems; EoLC, end-of-life care; PPD, preferred place of death.

**Table 4:** Characteristics of EPaCCS studies reporting hospital admission

Author	Sample	% with cancer	Likelihood of being admitted to hospital OR (95% CI)*	Likelihood of attend A&E OR (95% CI)†
Ali *et al* 28	401	100	0.41 (0.24 to 0.71)‡	
Purdy *et al* 34	1022§	68	0.65 (0.33 to 1.30)¶	0.57 (0.29 to 1.11)¶
	2572**		0.41 (0.28 to 0.60)¶	0.61 (0.40 to 0.92)¶

*OR for being admitted to hospital for those with an EPaCCS record versus those without an EPaCCs record.

†OR for having an A&E visit in the last 30 days of life for those with an EPaCCS record versus those without an EPaCCS.

‡Adjusted for reason for attending the emergency department.

§North Somerset.

¶Adjusted for gender, age, deprivation and condition.

**Somerset.

A&E, Accident and Emergency Department; EPaCCS, electronic palliative care coordination systems.

### Description of studies

#### Expert opinion articles

Fourteen articles were classified as expert opinion. Two were editorials,^16 20^ nine were commentaries,^15 17–19 21 23 25–27^ two were discussion and analytical papers,^5 24^ and one was a narrative review on advance care planning.^22^ Five articles provided an overview of EPaCCS in the UK context, including implementation challenges and references to data from audits, internal reports and some published articles.^5 17 18 21 24^ Six articles were a commentary to another published study.^15 19 23 25–27^ In four commentary articles and one editorial, authors expressed a favourable opinion regarding the potential of EPaCCS to improve end-of-life care outcomes.^17 18 20 23 26^ Three commentaries raised concerns about the lack of strong evidence for EPaCCS and the difficulties and potential bias of the current available research.^15 25 27^ Lindsey and Hayes^21^ provided an overview of EPaCCS, including definitions, concepts and mandatory elements required to develop and implement them in the UK.

Riley and Madill^24^ discussed the experience of developing and implementing an EPaCCS in London.^24^ The article provided preliminary audit data, and described how EPaCCS might impact on end-of-life care outcomes, providing some experiential examples. Petrova *et al* 5 present a critical analysis of EPaCCS. The article discussed five key challenges for EPaCCS: the need to involve different actors and settings, the complexity of an intervention that requires a change in the culture of an organisation, the lack of interoperability of IT systems in the UK, information governance issues, and uncertainties and sensibilities regarding end-of-life issues. The authors proposed six drivers for the successful spread of EPaCCS and the next steps for improvements.

#### Quantitative articles

Of the nine quantitative articles, seven reported an aspect of patients’ management such as place of death or hospital admission,^6 7 28–30 32 34^ and three reported an aspect of the process of implementing EPaCCS.^6 31 33^

##### Place of death and achieving preferred place of death

Six articles were focused on the place of death.^6 7 29 30 32 34^ These included five retrospective cohort studies,^6 7 29 30 34^ and one cross-sectional study.^32^

Five articles reported the preferred place of death of people with an EPaCCS record,^6 7 29 30 32^ of which four reported the proportion of patients who achieved their preference.^6 7 29 30^ Dying at home was preferred by 35% to 65% of patients, and 55% to 79% of individuals with an EPaCCS record achieved their preference (table 3). Four articles examined factors associated with achieving preferred place of death, among people with EPaCCS records. In Callender *et al*, being female, having cancer as primary diagnosis, a WHO performance score of 4, a not for resuscitation status and reporting hospice or care home as preferred place of death were associated with increased odds of achieving the preferred place of death.^30^ Allsop *et al* 6 found that patients who reported hospice or care homes as preferred place of death were more likely to have achieved their preferred place of death. Smith *et al* 7 reported that a smaller proportion of patients that preferred to die in a hospice achieved their preferred place of death compared with patients who preferred to die at home or in a care home. Broadhurst *et al* 29 found that individuals with an EPaCCS who also had a ceiling of treatment plan recorded were more likely to die in their preferred place of death. None of these studies compared people with EPaCCS records with those without an EPaCCS record.

Purdy *et al* 34 carried out a retrospective cohort study with control group. They identified individuals who were potentially eligible for end-of-life care and had died between 2011 and 2012, from healthcare records. They found individuals with an end-of-life electronic record had lower odds of death in hospital than individuals without an end-of-life electronic record after adjusting by gender, age, deprivation and cause of death (table 3). In this study, EPaCCS were implemented as part of a complex intervention that included improved out-of-hours provision and coordination between hospital and community settings, and only 12% of the eligible sample had an EPaCCS record.

### Admission to hospital

Two articles focused on hospital admissions^28 34^; both were retrospective cohort studies with control groups.

Ali *et al* 28 report a retrospective cohort study among individuals with cancer in Grampian. Electronic records were searched for individuals with a consultation related to a cancer diagnosis and terminal care. The content of consultation was manually reviewed to confirm the cancer diagnosis. No information on type or stage of cancer was reported. The authors identified the presence or absence of an EPaCCS record. They found the likelihood of being admitted to hospital within a year for patients with a diagnosis of advanced cancer was lower in individuals registered on the EPaCCS system compared with individuals without an EPaCCS record (table 4). However, the authors reported that implementation of EPaCCS was limited by difficulties uploading summaries into the system.

Purdy *e* *t al* 34 found that the chances of being admitted to hospital and having emergency department attendance in the last 30 days of life were lower in individuals with advanced life-threatening illnesses who had an EPaCCS compared with those who did not (table 4).

### Use of EPaCCS

Three articles focused on use of EPaCCS: one retrospective cohort study,^6^ one cross-sectional study^31^ and one service evaluation.^33^ Two articles reported the proportion of patients with EPaCCS in the general population,^6^ and in a palliative care population,^33^ and found EPaCCS were used in 27% and 54% of patients in these groups, respectively.

Millares Martin surveyed 209 CCGs in England regarding the level of EPaCCS implementation in their area. Nationally, 29% of CCGs had no EPaCCS in use, and the proportion of CCGs with a fully operative EPaCCS varied from 34% in the Midlands and East of England to 84% in London.^31^

### Qualitative and mixed-methods articles

The three qualitative (one of which was mixed-methods) articles focused on how different EPaCCS systems were implemented in practice, and the challenges of developing and implementing an EPaCCS system locally or nationally.^35–37^ Common challenges included difficulties with IT systems and poor uptake among primary care and in-hours staff.

Hall *et al* 35 described a new EPaCCS system in Scotland and explored both perceptions and feasibility of its implementation. Views of patients, carers, primary care and out-of-hours staff were positive and thought to be beneficial to patients, while out-of-hours staff identified that it supported and facilitated their work. Key issues that arose centred on technical problems and poor uptake within the primary care setting.

Wye *et al* 36 37 presented data from a service evaluation of an innovative palliative care programme that included a new EPaCCS in Southwest England. Wye *et al* 36 found that use of the system was often patchy and perceived to work best for those people with cancer who had fast track funding in place and who were close to death. They also highlighted that programme’s success was in part due to their dedicated and motivated staff. Wye *et al* 37 identified that out-of-hours staff found EPaCCS records useful, however, in-hours staff perceived EPaCCS records to be problematic as they meant additional work with little or no benefit for them. Record access was also very poor among paramedics, which was thought to be mainly due to technical issues. This study included quantitative data on place of death and likelihood of dying in hospital, from the same cohort analysed by Purdy *et al*.^34^

### Reports

The Ipsos MORI report was an independent evaluation of the EPaCCS pilot programme. The report described variation in the level of implementation and challenges that the eight pilot sites experienced, based on views and experiences of stakeholders. Data from the pilot sites indicated EPaCCS were well received by patients and carers. Stakeholders reported benefits including improved communication, delivery of patient choices and reduction in unnecessary admissions or appointments. Concerns were expressed regarding data sharing and security, increase in GP workload, the process of patient consent, engaging in difficult end-of-life conversations, ensuring the inclusion of non-cancer patients, integrating data from multiple IT systems, the development of multiple registers systems across the UK and the timely access of all providers to the register.^39^

National Health Service (NHS) Improving Quality performed an economic evaluation based on four early implementer sites. The report analysed aggregated data from Hospital Episode Statistics and Office of National Statistics before and after EPaCCS implementation, and found use of EPaCCS was associated with an increase in the proportion of people dying in their usual place of residence, and a small reduction in the number of unscheduled admissions and length of stay in hospital. No statistics were performed to understand whether these differences were significant or not.^40^ The report found 63% to 77% of individuals with an EPaCCS died in their preferred place, and 6% to 18% of individuals with an EPaCCS died in hospital. The report concluded that EPaCCS could potentially generate savings from £399 to £1480 per death avoided in hospital.^40^

The National End of Life Care Intelligence Network carried out a survey of 211 CCGs in 2013. Sixty-four CCGs (30%) reported having an operational EPaCCS in place. A total of 111 (53%) had started planning for EPaCCS implementation, and 10 (5%) had no plans. Ten different technical systems of EPaCCS were reported by CCGs and only 15 CCGs reported full compliance with all the items included in the national information standard. Only 17% of CCGs had accurate information regarding the actual place of death and death in the preferred place for individuals on EPaCCS.^38^

A more recent evaluation developed by the Whole Systems Partnership collected information from five sites with established EPaCCS and six control sites, and included in-depth interviews of patients, carers and healthcare professionals.^41^ The interviews identified challenges including identifying who should have an EPaCCS record, difficulties of having advance care planning conversations, the need for education and training in end-of-life care choices, ensuring records are updated and the time needed to complete an EPaCCS. In general, patients and healthcare professionals felt that the system worked well the majority of the time, and that EPaCCS helped patients avoid repeating information. The evaluation compared aggregate data on place of death and hospital admissions in CCGs with and without EPaCCS, and reported no difference in the proportion of people dying in their usual place of residence, or in the proportion of hospital deaths, hospital admissions or costs of care in the last year of life.^41^

## Discussion

This systematic review of the state of the science of EPaCCS shows that most of the current evidence comes from expert opinion and observational studies, and that there is an absence of any studies with an experimental design evaluating the impact of EPaCCS. While observational studies focused on place of death, hospital utilisation and use of EPaCCS, qualitative studies were mainly focused on the challenges of EPaCCS design and implementation.

The quantitative studies identified were either retrospective or cross-sectional in design, and focused on processes of care before death: the place of death of people with an EPaCCS record, the frequency of hospital admission and EPaCCS use. The proportion of patients with an EPaCCS record who died in their preferred place varied from 55% to 79%,^6 7 29 30^ which was noted to be higher than average for the population. The highest level of evidence came from two retrospective cohort studies that compared people with and without EPaCCS records, and found EPaCCS use was associated with lower odds of hospital death, hospital admission and emergency department attendance.^28 34^ Both these studies note important limitations, including lack of information on potential confounders such as comorbidities and stage of disease. While EPaCCS might contribute to an increase in the number of people who die at home, it is also possible that individuals who are included in these records are those who are actively seeking, and therefore are more likely, to die at home. It is not possible to rule out this selection bias in the group of people who have EPaCCS records due to the lack of randomisation within the studies. Indeed, the mixed-methods study by Wye *et al* explored the relationship between EPaCCS and home death and found that EPaCCS were almost exclusively used by GPs and community nurses, which might explain their strong association with home death.^37^

The qualitative studies identified, as well as some of the reports, suggest that EPaCCS are acceptable for patients and healthcare professionals.^35 36 39 41^ However, there were some discrepancies between perceptions of benefits and utility expressed by different health professionals. In-hours staff perceived EPaCCS as a potential burden due to an increased workload without perceivable benefit to them, while out-of-hours staff perceived EPaCCS to be more useful.^37^ Although patients’ perspectives and opinions about EPaCCS were included in two of the articles,^35 36^ the majority of the results and recommendations from these studies were based on healthcare professionals’ views.^35–37^ More insight into patient and families’ experiences of EPaCCS is needed.

We found a dearth of information on patient outcomes or quality of life measures. None of the studies we identified explicitly explored the potential harms of using EPaCCS, though from the healthcare professional’s perspective some articles discussed the burden of inputting data, and data-sharing issues, as a potential problem.^35 37^ From the patient’s perspective, Wye *et al* 37 suggest that once created, records are infrequently updated and it is unclear if this can have an impact on patients who change their preferences as their disease progresses. There is considerable evidence that suggests people prefer to die at home.^42^ However, some hospital admissions near the end of life are appropriate, particularly where prognosis is uncertain. The potential for harm through avoidance of appropriate hospital care should be explored.

Two reports explored the economic impact of EPaCCS, with conflicting results.^40 41^ Based on a before–after design using area-level data, the NHS Improving Quality report found implementation of EPaCCS was associated with a reduction in hospital deaths that could potentially generate savings of £399–£1480 per hospital death avoided. In contrast, the Whole Systems Partnership compared data on place of death and hospital admissions in CCGs with and without EPaCCS, and found no difference in the proportion of people dying in their usual place of residence, or in the proportion of hospital deaths, hospital admissions or costs of care in the last year of life. Both studies had limitations, including a lack of adjustment for confounders and consideration of only the direct costs involved. Further studies of the economic impact of EPaCCS are needed.

### Strengths and limitations

Our study was motivated by the increasing political focus on EPaCCS as a tool to improve end-of-life care in the UK, and the lack of clarity regarding the underlying evidence base for these policies. In the preliminary searches, we found that the non-UK literature described tools where coordination between healthcare settings was not the primary focus. Therefore, we included only articles describing UK EPaCCS. Research from other settings might provide relevant information to guide implementation and use of EPaCCS. While our review necessarily included only UK data, there are important implications more broadly in terms of understanding the evidence base for policy interventions.

We did not involve directly an information specialist in the search design, as is suggested for systematic reviews, which could potentially have affected the sensitivity of our search strategy. We searched a limited number of databases, which might have led to exclusion of relevant articles. However, it is unlikely that any study with an experimental design could have been missed.

As our aim was to understand the state of the science of EPaCCS, we included all relevant published articles. This inclusive approach contributes to the strengths of our study, for example, by highlighting that 14 of 26 articles published are expert opinion, and a dearth of information on patient outcomes. We used the Standard Quality Assessment Criteria to appraise the articles included.^11^ Appraisal of both qualitative and quantitative studies using a common tool is challenging, we addressed this by using two authors to independently rate the studies and discussion with a third where there were disagreements. The inclusion of government and independent reports in this review adds to its depth, and highlights the fact that much evidence for EPaCCS is located within grey literature. Other areas of grey literature, for example, unpublished data, were not systematically identified.

### Implications

While innovation to improve the quality of care received by patients is highly desirable, widely promoting interventions in the absence of strong supporting evidence can be dangerous. Logical and well-intentioned policy recommendations can do more harm than good.^43^ The studies described in this systematic review highlight the important potential benefits of EPaCCS for improving the end-of-life care. However, observational studies can overestimate the effect of interventions,^44^ and the lack of strong evidence is of concern. All interventions may have benefits and harms, some of which are more predictable than others.^45^ More and urgent research is needed in order to fully understand the benefits and potential harms of EPaCCS. Evaluations using experimental or quasi-experimental designs are needed, and these should be carried out alongside further qualitative studies to understand what needs to be in place to maximise benefits and avoid harms.^46^ Given the current drive for national roll-out of EPaCCS by 2020, it is essential that rigorous evaluation of EPaCCS is prioritised.
