# Supplementary material for: Electronic palliative care coordination systems (EPaCCS): a systematic review
Source: BMJ Support Palliat Care. 2019 May 8;10(1):68–78. doi: 10.1136/bmjspcare-2018-001689 (PMC7030943; doi:10.1136/bmjspcare-2018-001689)
Supplement: Supplementary data [file bmjspcare-2018-001689supp001.pdf]

Supplementary File 1.

Search strategy by database searched

Medline

1. EPaCC*.mp.
2. coordinate my care.mp.
3. my care choices.mp.
4. share my care.mp
5. Partnership for Excellence in Palliative Support project.mp.
6. electronic KIS.mp
7. end of life care regist*.mp
8. eol* regist*.mp
9. electronic key information summar*.mp
10. electronic palliative care summar*.mp
11. electronic palliative care system*.mp
12. Electronic palliative care coordination system*.mp
13. 1 or 2 or 3 or 4 or 5 or 6 or 7 or 8 or 9 or 10 or 11 or 12
14. exp Medical Records Systems, Computerized/
15. exp Electronic Health Records/
16. electronic record*.mp
17. ((electronic or automat* or digital or on-line or computer) adj2 (health or medical or personal or patient) adj2 (record* or registr* or system*)).mp
18. 14 or 15 or 16 or 17
19. exp Advance Care Planning/
20. exp Advance Directives/
21. exp Living Wills/
22. advance* care plan*.mp
23. (advance* adj2 (directive* or care or decision*)).mp
24. living will*.mp
25. 19 or 20 or 21 or 22 or 23 or 24
26. 18 and 25
27. 13 or 26

Embase

1. EPaCC*.mp.
2. coordinate my care.mp.
3. my care choices.mp.
4. share my care.mp.
5. Partnership for Excellence in Palliative Support project.mp.
6. electronic KIS.mp.
7. end of life care regist*.mp.
8. eol* regist*.mp.
9. electronic key information summar*.mp.
10. electronic palliative care summar*.mp.
11. electronic palliative care system*.mp.
12. Electronic palliative care coordination system*.mp.
13. 1 or 2 or 3 or 4 or 5 or 6 or 7 or 8 or 9 or 10 or 11 or 12
14. exp Medical Records Systems, Computerized/
15. exp Electronic Health Records/
16. exp electronic patient record/
17. electronic record*.mp.
18. ((electronic or automat* or digital or on-line or computer) adj2 (health or medical or personal or patient) adj2 (record* or registr* or system*)).mp.
19. 14 or 15 or 16 or 17 or 18
20. exp Advance Care Planning/
21. exp Advance Directives/
22. exp Living Wills/
23. advance* care plan*.mp.
24. (advance* adj2 (directive* or care or decision*)).mp.
25. living will*.mp.
26. 20 or 21 or 22 or 23 or 24 or 25
27. 19 and 26
28. 13 or 27

Cinahl

S1	TI EPaCC* OR AB EPaCC*
S2	TI coordinate my care OR AB coordinate my care
S3	TI my care choices OR AB my care choices
S4	TI share my care OR AB share my care
S5	TI Partnership for Excellence in Palliative Support project OR AB Partnership for Excellence in Palliative Support project
S6	TI electronic KIS OR AB electronic KIS
S7	TI end of life care regist* OR AB end of life care regist*
S8	TI eol* regist* OR AB eol* regist*
S9	TI electronic key information summar* OR AB electronic key information summar*
S10	TI electronic palliative care summar* OR AB electronic palliative care summar*
S11	TI electronic palliative care system* OR AB electronic palliative care system*
S12	TI Electronic palliative care coordination system* OR AB Electronic palliative care coordination system*
S13	MH electronic health records
S14	MH medical record linkage
S15	MH patient record systems
S16	TI ((electronic* or automat* or on-line or digital or computer) n2 (health or medical or personal or patient) n2 (record* or registr* or system)) OR AB ((electronic* or automat* or on-line or digital or computer) n2 (health or medical or personal or patient) n2 (record* or registr* or system))
S17	MH advance care planning
S18	MH advance directives
S19	MH living wills
S20	TI living will* OR AB living will*
S21	TI advance* care plan* OR AB advance* care plan*
S22	TI ((advance* n2 (directive* or care or decision))) OR AB ((advance* n2 (directive* or care or decision)))
S23	S17 OR S18 OR S19 OR S20 OR S21 OR S22
S24	TI electronic record* OR AB electronic record*
S25	S13 OR S14 OR S15 OR S16 OR S24
S26	S1 OR S2 OR S3 OR S4 OR S5 OR S6 OR S7 OR S8 OR S9 OR S10 OR S11 OR S12
S27	S23 AND S25
S28	S26 OR S27

Cochrane Library

#1 (epacc*):ti,ab,kw

#2 (coordinate my care):ti,ab,kw

#3 ("my care choices"):ti,ab,kw

#4 ("share my care"):ti,ab,kw

#5 ("Partnership for Excellence in Palliative Support project"):ti,ab,kw

#6 ("electronic KIS"):ti,ab,kw

#7 (eol* NEXT regist*):ti,ab,kw

#8 ("end of life care" next regist*):ti,ab,kw

#9 ("electronic key information" next summar*):ti,ab,kw

#10 ("electronic palliative care" next summar*):ti,ab,kw

#11 ("electronic palliative care" next system*):ti,ab,kw

#12 ("electronic palliative care coordination system"):ti,ab,kw

#13 #1 or #2 or #3 or #4 #5 or #6 or #7 or #8 or #9 or #10 or #11 or #12

#14 MeSH descriptor: [Electronic Health Records] explode all trees

#15 MeSH descriptor: [Medical Records Systems, Computerized] explode all trees

#16 (electronic next record*):ti,ab,kw

#17 ((electronic or automat* or digital or on-line or computer) near/2 (health or medical or personal or patient) near/2 (record* or registr* or system*)):ti,ab,kw

#18 #14 or #15 or #16 or #17

#19 MeSH descriptor: [Advance Care Planning] explode all trees

#20 MeSH descriptor: [Advance Directives] explode all trees

#21 MeSH descriptor: [Living Wills] explode all trees

#22 (advance* next care next plan*):ti,ab,kw

#23 (advance* near/2 (directive* or care or decision*)):ti,ab,kw

#24 (living next will*):ti,ab,kw

#25 #19 or #20 or #21 or #22 or #23 or #24

#26 #18 and #25

#27 #13 or #26
