# Supplementary material for: Electronic palliative care coordination systems (EPaCCS): a systematic review
Source: BMJ Support Palliat Care. 2019 May 8;10(1):68–78. doi: 10.1136/bmjspcare-2018-001689 (PMC7030943; doi:10.1136/bmjspcare-2018-001689)
Supplement: Supplementary data [file bmjspcare-2018-001689supp002.pdf]

Supplementary File 2

Table 1. Template for data extraction.

**First author**	Surname Name
**Year**	year of publication
**Journal**	Journal of publication
**Qual/Quant**	Was the study a qualitative study, quantitative study or both
**Level of evidence**	Was the study exploring an outcome related with the patient or a process outcome
**Outcome classification**	Achievement of PPD, Admission to hospital or length of stay, implementation or ACP documentation.
**Study design**	type of study design
**Aim of the study**	copy and paste from publication
**Setting**	Centre or place where the study was performed
**Participants**	Number of participants and characteristics
**Outcome**	Primary and secondary outcomes of the study
**Results**	Results for the outcomes mentioned
**Comments**	

Table 2. Excluded studies and reason for exclusion.

Study	Reason for exclusion
Brothers H. et al, 2015	not about electronic records
Burridge EJ. et al, 2016	not about electronic records
Caners K. et al, 2015	The electronic system was not focus on coordination and sharing of information
Cawley D. et al, 2014	Conference abstract
Collis E. et al, 2016	Conference abstract
Coulthard P. et la, 2015	not about electronic records
Daniel C. & Choquet R., 2015	The electronic system was not focus on coordination and sharing of information
Dickinson GE. et al, 2008	not about electronic records
Ding JJ. et al, 2018	not about electronic records
Doherty L., 2008	not about electronic records
Etkind SN. et al, 2014	Not about ACP
Falconer EK & Docherty JP., 2018	Not about ACP
Finucane A. et al, 2016	Conference abstract
Flaherty CF. et al, 2018	not about electronic records
Garden G. et al, 2018	The electronic system was not focus on coordination and sharing of information
Henry K. et al, 2014	Conference abstract
Holms N. et al, 2014	not about electronic records
Huber M. et al, 2018	The electronic system was not focus on coordination and sharing of information
Islam MM. et al, 2018	The electronic system was not focus on coordination and sharing of information
Jenkins R., 2012	not about electronic records
Johnstone R. et al, 2011	not about electronic records
Karbasi C. et al, 2018	not about electronic records
Khan-Mahmood K. et al, 2016	Conference abstract
Kitzinger CC., 2010	not about electronic records
Kwakwa J. et al, 2014	Conference abstract
Lemon C. et al., 2018	The electronic system was not focus on coordination and sharing of information
Moore A. et al, 2007	The electronic system was not focus on coordination and sharing of information
Murray C. et al, 2010	The electronic system was not focus on coordination and sharing of information
Murray-Brow F., 2014	Conference abstract
Onaç R. et al, 2010	The electronic system was not focus on coordination and sharing of information
Patkar V. et al, 2010	Not about ACP
Petrova M. & Barcley S., 2016	Conference abstract
Petrova M. et al, 2014	Conference abstract
Pini S. et al, 2018	not about electronic records
Riley J & Smith CF., 2010	Conference abstract
Rose B. et al, 2016	The electronic system was not focus on coordination and sharing of information
Sayma M. et al, 2018	not about electronic records

Shahmoradi L. et al, 2017	not about electronic records
Singh J. et al, 2013	not about electronic records
Spears J. et al, 2017	not about electronic records
Thomas C. et al, 2018	Conference abstract
Vassallo et al, 2013	not about electronic records
Velikova et al, 2012	not about electronic records
White N., 2017	not about electronic records
Wiggins N. et al, 2018	Conference abstract
Department of Health. End of Life Care Strategy: Fourth Annual Report. Oct 2012	Not specific about EPaCCS
Department of Health. End of Life Care Strategy - promoting high quality care for all adults at the end of life. 2008	Not specific about EPaCCS
National End of Life Care Programme. Making the case for change: electronic Palliative Care Co-ordination Systems. Oct 2012	Duplicate sample
National End of Life Care Programme. Electronic Palliative Care Co-ordination Systems (EPaCCS) Mid 2012 survey report. Oct 2012	Duplicate sample
NHS improving quality. Implementing an Electronic Palliative Care Co-ordination System (EPaCCS). Jul 2005	Duplicate sample
NHS England. Actions for end of life care: 2014-16. Nov 2014	Not specific about EPaCCS
National End of Life Care Intelligence Network (NEoLCIN). Palliative care co-ordination: core content. Implementation guidance. Sept 2015	Not specific about EPaCCS

Table 3. Quality appraisal of quantitative included studies.

	Question / objective sufficiently described?	Study design evident and appropriate?	Method of subject/comparison group selection or source of information/input variables described and appropriate?	Subject (and comparison group, if applicable) characteristics suffi ciently	If interventional and random allocation was possible, was it described?	If interventional and blinding of investigators was possible, was it reported?	If interventional and blinding of subjects was possible, was it reported?	Outcome and (if applicable) exposure measure(s) well defined and robust to measurement / misclassification bias? Means of assessment reported?	Sample size appropriate?	Analytic methods described/ justified and appropriate?	Some estimate of variance is reported for the main results?	Controlled for confounding?	Results reported in sufficient detail?	Conclusions supported by the results?	Total	N/A	SCORE
Ali A. et al., 2013	1	1	2	2	n/a	n/a	n/a	2	2	1	2	1	2	2	18	3	81.8
Allsop M.J. et al, 2017	1	1	2	2	n/a	n/a	n/a	2	n/a	n/a	n/a	n/a	1	1	10	7	71.4
Broadhurst HL. et al, 2018	2	2	2	2	n/a	n/a	n/a	2	2	1	2	0	2	2	19	3	86.4
Callender T. et al, 2017	2	2	2	2	n/a	n/a	n/a	2	2	2	2	2	2	2	22	3	100.0
Millington-Sanders C. et al, 2013	2	1	2	1	n/a	n/a	n/a	2	n/a	n/a	n/a	n/a	2	2	12	7	85.7
Millares Martin P., 2018	2	2	1	2	n/a	n/a	n/a	1	2	2	n/a	n/a	1	1	14	5	77.8
Pringle A. et al, 2014	1	2	2	1	n/a	n/a	n/a	2	n/a	n/a	n/a	n/a	2	2	12	7	85.7
Purdy S. et al, 2014	2	2	2	2	n/a	n/a	n/a	2	2	2	2	1	2	2	21	3	95.5
Smith C. et al, 2012	1	1	2	2	n/a	n/a	n/a	2	n/a	n/a	n/a	n/a	1	2	11	7	78.6

Table 4. Quality appraisal of qualitative included studies.

	Question / objective sufficiently described?	Study design evident and appropriate?	Context for the study clear?	Connection to a theoretical framework / wider body of knowledge?	Sampling strategy described, relevant and justified?	Data collection methods clearly described and systematic?	Data analysis clearly described and systematic?	Use of verification procedure(s) to establish credibility?	Conclusions supported by the results?	Reflexivity of the account?	TOTAL	SCORE
Hall S. et al, 2012	2	2	1	0	2	2	1	2	2	1	15	75.0
Wye L. et al, 2014	2	2	2	2	2	2	2	2	2	1	19	95.0
Wye L. et al, 2016	2	2	1	0	2	2	2	2	2	1	16	80.0
